# Dipole Density Guided Catheter Ablation versus Conventional Substrate Modification for Repeat Catheter Ablation of Persistent Atrial Fibrillation

**DOI:** 10.3390/jcm13010223

**Published:** 2023-12-30

**Authors:** Jan-Hendrik Schipper, Daniel Steven, Jakob Lüker, Jonas Wörmann, Jan-Hendrik van den Bruck, Karlo Filipovic, Sebastian Dittrich, Cornelia Scheurlen, Susanne Erlhöfer, Friederike Pavel, Arian Sultan

**Affiliations:** Department of Electrophysiology, Heart Center, University of Cologne, Kerpener Str. 62, 50937 Cologne, Germany; daniel.steven@uk-koeln.de (D.S.); jakob.lueker@uk-koeln.de (J.L.); jonas.woermann@uk-koeln.de (J.W.); jan-hendrik.van-den-bruck@uk-koeln.de (J.-H.v.d.B.); karlo.filipovic@uk-koeln.de (K.F.); sebastian.dittrich1@uk-koeln.de (S.D.); susanne.erlhoefer@uk-koeln.de (S.E.); friederike.pavel@uk-koeln.de (F.P.); arian.sultan@uk-koeln.de (A.S.)

**Keywords:** dipole density, atrial fibrillation, catheter ablation, persistent atrial fibrillation, pulmonary vein isolation

## Abstract

Aims: The optimal ablation strategy for recurrent persistent atrial fibrillation (persAF) after initially successful catheter ablation (CA) remains debatable. Dipole density (DD) guided CA using the AcQMap system has been proven to be feasible and effective in patients with persAF. So far, long-term outcome data for DD-guided CA in patients with recurrence of persAF are sparse. This study sought to assess long-term outcome data in patients undergoing a DD-guided CA for recurrence of persAF after previous CA in comparison to conventional repeat CA. Methods and Results: Patients undergoing DD-guided CA for recurrence of persAF after previous ablation were compared to patients undergoing conventional substrate modification (CSM). A total of 64 patients (32 DD-guided and 32 CSM) were included in this analysis. Procedure duration (DD: 236 ± 61 min; CSM: 198 ± 59 min; *p* = 0.004) and fluoroscopy time (DD: 36 ± 15 min; CSM: 20 ± 11 min; *p* = 0.0001) were significantly longer in the DD group. After a long-term median follow-up (FU) of 27 months (interquartile range 12.8–34.3), DD-guided CA was inferior to CSM regarding overall arrhythmia-free survival (DD: 6 patients (19%), CSM: 11 patients (34%); HR 1.47; *p* = 0.04). Freedom from AF did not differ between both groups (DD: 16 patients (50%); CSM: 18 patients (56%), HR 0.99, *p* = 0.47). During FU, more patients underwent repeat CA after DD-guided ablation (DD: 16 patients (50%), CSM: 7 patients (22%), *p* = 0.04). No major complications occurred overall. Conclusions: Dipole density-guided CA is equally safe but associated with longer procedure duration compared to conventional substrate modification for treatment of recurrent persAF after previous CA. Of note, long-term arrhythmia-free survival is significantly worse after DD-guided ablation, and more patients undergo redo procedures.

## 1. Introduction

In patients undergoing catheter ablation (CA) for persistent atrial fibrillation (persAF), previous studies have shown that pulmonary vein isolation (PVI) is only sufficient in a considerable proportion of patients [1,2].

If recurrence of persAF occurs after initially successful PVI, the optimal ablation approach remains debatable. In the pursuit of identifying the most effective strategy for persAF, various extensive ablation approaches have been investigated (LAA isolation [3], posterior wall isolation [4], MRI-detected fibrosis-guided ablation [5,6], vein of Marshall ethanol infusion [7], spatiotemporal electrogram dispersion ablation [8], electroanatomical guided ablation [9]). However, none of these techniques has yet emerged as the universally accepted gold standard. Newer mapping technologies such as rotor mapping and FIRM mapping [10,11] have been proposed to display AF mechanisms and potentially deliver more tailored ablation approaches but also failed to show superiority over PVI regarding arrhythmia-free survival [12].

Dipole density mapping represents a novel strategy displaying AF wavefronts and mechanisms guiding CA in persAF in a different manner [13,14]. It is based on the dipole density of the myocardium, quantifying the system’s polarity and is defined as cellular charge sources [15,16]. A DD-guided map depicts a more localized display of the electrical activation compared to a conventional voltage map [15]. So far, a non-randomized trial reported a potential benefit of DD-guided CA in patients undergoing repeat CA for persAF with a 12-month arrhythmia-free survival of 53% after a single procedure on and off antiarrhythmic drugs [17].

However, long-term outcome data for DD-guided repeat CA in patients with recurrence of persAF after initially successful CA are sparse. We sought to evaluate long-term outcome data and compare DD-guided CA to conventional substrate modification (CSM) in the setting of recurrent persAF after previous CA.

## 2. Materials and Methods

### 2.1. Study Population

This retrospective single-center study included 64 patients with recurrence of persAF undergoing repeat CA between June 2017 and January 2021. Only patients with at least one previous CA for persAF were included. We pair-matched 32 patients after DD-guided CA with 32 patients who underwent a conventional radiofrequency (RF) repeat CA regarding age, sex, body mass index (BMI), left atrial (LA) diameter, and the total number of previous CA for persAF. Data acquisition was conducted using an electronic data capture system (RedCap Database, Nashville, TN, USA).

The study was approved by the local Ethics Committee of the University of Cologne and complied with the Declaration of Helsinki. All patients provided written informed consent.

### 2.2. Procedures

In all procedures (DD-guided and conventional RF ablation), oral anticoagulation was interrupted on the day of CA and continued on the evening of the procedure. If patients received vitamin K antagonists, an international normalized ratio (INR) value of 2–3 was accepted for the procedure. In accordance with the latest AF guidelines, transesophageal echocardiography was performed prior to ablation if indicated [18]. The procedures were conducted in deep analgosedation using propofol, midazolam, and fentanyl. After establishing the femoral venous access, a decapolar reference catheter (Inquiry, Abbott, Abbott Park, IL, USA) was placed in the coronary sinus. Transseptal puncture (TSP) was performed under fluoroscopic guidance (Swartz Braided Transseptal Guiding Introducers and BRK Transseptal Needles, Abbott, Abbott Park, IL, USA). After TSP, a weight-adapted bolus of heparin followed by repetitive boli was administered to maintain an effective anticoagulation with an activated clotting time (ACT) > 300 s during the procedure.

In both groups, monitoring of the esophageal temperature was established using a temperature probe (S-Cath, Esophageal Temperature Probe, Circa Scientific Inc., Englewood, CO, USA). The procedure time was defined as the time from groin puncture to sheath removal (skin-to-skin time). A figure-of-eight suture for venous closure [19] with an additional compression badge (for 6 h) was applied after sheath removal. Directly after the procedure, pericardial effusion was ruled out by transthoracic echocardiography (TTE) in the electrophysiology (EP) laboratory. Repeat TTE examinations were performed 2 and 24 h after the procedure.

### 2.3. Dipole Density Guided Ablation

For DD-guided ablations, the AcQMap system (Acutus Medical, Carlsbad, CA, USA) was used. It is a non-contact high-resolution mapping system using a single array-shaped catheter providing 48 ultrasound probes for 3D anatomy reconstruction and 48 electrodes creating activation maps of electrical conduction based on DD displaying AF wavefronts [12]. Compared to conventional voltage mapping, farfield artifacts can be reduced because only the local-charge sources are displayed [16,20].

After obtaining LA access, the transseptal sheath was exchanged for a steerable sheath (12 F AcQGuide sheath, Acutus Medical, Carlsbad, CA, USA) and the AcQMap catheter (10 F, Acutus Medical, Carlsbad, CA, USA) was positioned in the LA. In patients who presented with SR at the start of the procedure, AF was induced by atrial burst pacing. A 3D ultrasound-based anatomy of the LA was created using the 48 ultrasound probes of the AcQ-Map catheter, followed by obtaining a non-contact AF activation map.

Obtained AF propagation maps were then displayed on the 3D LA shell, highlighting pivotal ablation target sites and detecting three different pre-defined activation patterns in AF: focal, rotational, and irregular activation [20]. In the case of reconnected PVs, re-isolation was performed.

Assumed AF drivers were ablated using an irrigated tip non-contact force catheter (system prerequisite at the time of study) (Thermocool, Biosense Webster, Irvine, CA, USA) with a maximum power delivery of 30 W. The resulting ablation sites were connected to the next non-conducting anatomic barrier, if in the vicinity, to avoid the creation of new reentrant isthmuses. Ablation was continued until all displayed sites were thoroughly ablated, and respectively, all local signals were diminished. If further ablation at a specific region was futile or AF did not terminate, a DD re-map was obtained, and further ablation was performed. These steps were repeated until the termination of AF into SR or atrial tachycardia (AT). In case of the futility of further CA, the patient was converted into SR using electrical cardioversion. When AF terminated into AT, re-mapping, entrainment maneuvers, and ablation of AT was performed to achieve SR. After restoration of SR, isolation of the PVs and non-excitability of the ablation sites were assessed. In the case of lesion sets, lines were evaluated for a bidirectional block.

### 2.4. Conventional Substrate Modification

In all patients in the conventional arm, a 3D mapping system (CARTO3, Biosense Webster, Irvine, CA, USA and EnSite, Abbott, Abbott Park, IL, USA) was used to obtain LA anatomy and scar. Conventional CA was performed using RF energy applied by a contact-force catheter (Thermocool Smarttouch, Biosense Webster, Irvine, CA, USA and TactiCath, Abbott, Abbott Park, IL, USA) with a maximum power delivery of 40 W. High-power short-duration ablation (70 W) was performed in two patients. The ablation extent was at the operator’s discretion and was determined by low-voltage areas (<0.5 mV) and scar tissue (<0.2 mV) identified by the electroanatomical 3D map.

Two strategies were pursued: defragmentation of complex fractionated atrial electrograms (CFAE) (visually identified by the operator based on previously published criteria [21]) and/or anatomical linear ablations in the LA, including roof line, anterior line, mitral isthmus line and posterior wall isolation (PWI). The procedural endpoint was the non-excitability of the ablation sites and, in the case of linear ablations, the proof of bidirectional block using differential pacing and obtaining a propagation map during LAA pacing.

### 2.5. Follow-Up

Follow-up was obtained during outpatient clinic visits at 3 and 12 months after CA. Before every visit, a Holter ECG was performed to detect arrhythmia recurrence. During outpatient clinic visits, a 12-lead ECG was taken. Furthermore, tele-consultations and interrogation of cardiac implantable electronic devices (CIED) complemented the follow-up if applicable.

### 2.6. Endpoints

Recurrence of AF, AT, or atrial flutter lasting longer than 30 s after the blanking period of 90 days or a repeat CA were considered as the primary endpoint. Secondary endpoints consisted of the procedure and fluoroscopy time, total applied energy, RF duration, and the amount of high-frequency impulses.

Procedural associated complications (cardiac tamponade, major groin bleeding, transient ischemic attack (TIA), stroke or device-associated events, aspiration pneumonia) were the safety endpoints of this study.

### 2.7. Statistical Analysis

Continuous variables are presented as mean ± standard deviation, and categorical variables are summarized as counts and percentages. The D’Agostino–Pearson test was performed to test the normality of the data. Student’s *t*-tests were used for continuous variables if they were normally distributed. Otherwise, Mann–Whitney U-tests were performed. Fisher’s exact test was created in the case of dichotomous variables. Kaplan–Meier estimators were assessed for the outcome analysis. Here, the Gehan–Breslow–Wilcoxon test for the *p*-value and the Mantel–Haenszel test for hazard ratio were conducted. Statistical analyses were performed using Microsoft Excel (Version 16.80 for Mac, Microsoft Corporation, Redmond, WA, USA) and GraphPad Prism (Version 9.1.0 for Mac, GraphPad Software, San Diego, CA, USA). Statistical significance was defined as *p* < 0.05.

## 3. Results

### 3.1. Study Cohort

In this retrospective analysis, 64 patients (mean age: 65 ± 10 years, 48 (75%) male) with recurrence of persAF undergoing repeat CA after previous CA were analyzed. Baseline characteristics, comorbidities, and number of previous AF ablations did not differ between both groups (Table 1).

### 3.2. Procedural Characteristics

Analysis of AF patterns in the DD group revealed an irregular mechanism as the most prevalent AF pattern and could be detected in 19 (59%) patients, followed by focal (9 patients (28%)) and rotational (8 patients (25%)) mechanisms. On average, five re-maps were obtained (range 3–10). In 5 of 29 patients (17%) with AF, termination to SR was achieved through DD-guided CA. In 11 patients (38%), AF was terminated into AT, and then in 7 patients to SR. In 17 patients (53%), CV was necessary to obtain SR due to the futility of further ablation.

In the conventional group, isolation of the posterior wall (PWI) was performed significantly more often than in the DD group (DD: 2 patients (6%), CSM: 10 patients (31%), *p* = 0.02). In contrast, significantly more patients underwent defragmentation in the LA in the DD group (DD: 32 patients (100%), CSM: 23 patients (72%), *p* = 0.002). Other linear ablations are reported in Table 2.

The baseline rhythm at the start of the procedure was not different between both groups (Table 3). Procedure time (DD: 236 ± 61 min, CSM: 198 ± 59 min, *p* = 0.004) and fluoroscopy time (DD: 36 ± 15 min, CSM: 20 ± 11 min, *p* = 0.0001) were significantly longer in DD-guided CA compared to conventional CA. There were no significant differences regarding total applied energy during CA (DD: 123,553 ± 50,664 J; CSM: 121,138 ± 49,343 J, *p* = 0.98) and RF duration (DD: 4229 ± 1677 s; CSM: 3585 ± 1485 s, *p* = 0.16). However, the amount of high-frequency impulses (DD: 63 ± 28; CSM: 111 ± 77, *p* = 0.0004) was significantly lower in DD-guided CA. Procedural data are shown in Table 4.

### 3.3. Complications

Two patients (6%) after DD-guided CA suffered from postprocedural pneumonia due to aspiration and were treated with antibiotics compared to one patient (1%) after conventional substrate modification (*p* = 1.0). No other procedural-associated complications were reported. In the conventional group, 3 (9%) deaths occurred in the observation period due to non-procedural associated complications compared to none in the DD group (*p* = 0.24).

### 3.4. Outcomes

After a median follow-up of 27 months (interquartile range 12.8–34.3), 6 patients (19%) after DD-guided ablation were free from any atrial arrhythmia (AA) as opposed to 11 patients (34%) after conventional CA (*p* = 0.26). Freedom from AF was achieved in 16 patients (50%) after DD-guided CA and 18 patients (56%) after conventional CA (*p* = 0.80). During follow-up, AT occurred in 12 patients (38%) in the DD group and 11 patients in the CSM group (34%) (*p* = 1.0, Table 5).

**Figure 1:**
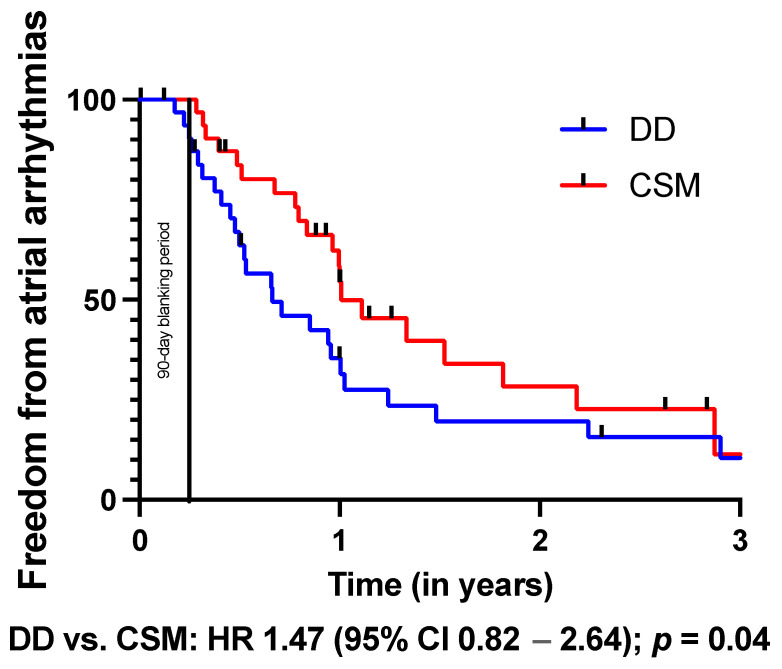
Kaplan–Meier analysis of freedom from atrial arrhythmias on or off AADs. A 90-day blanking period was observed.

**Figure 2:**
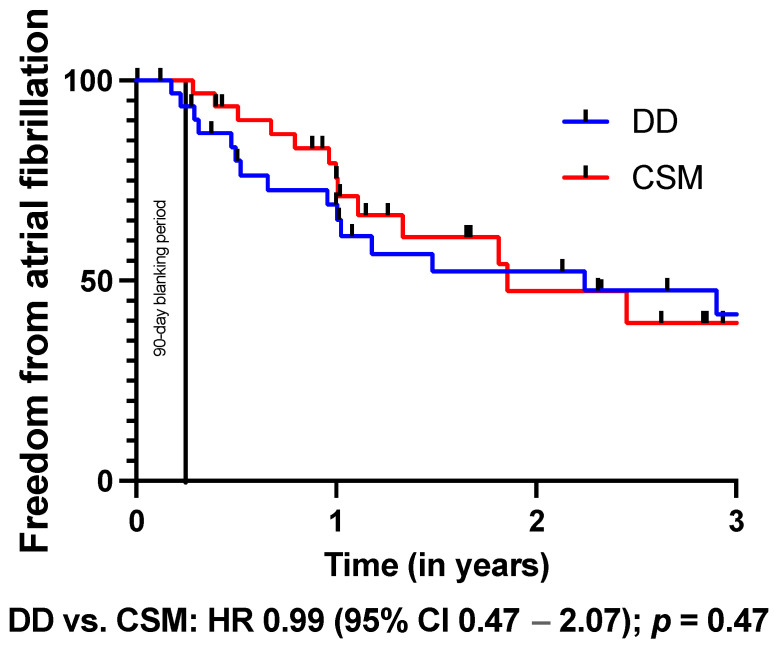
Kaplan–Meier analysis of freedom from atrial fibrillation on or off AADs. A 90-day blanking period was observed.

**Figure 3:**
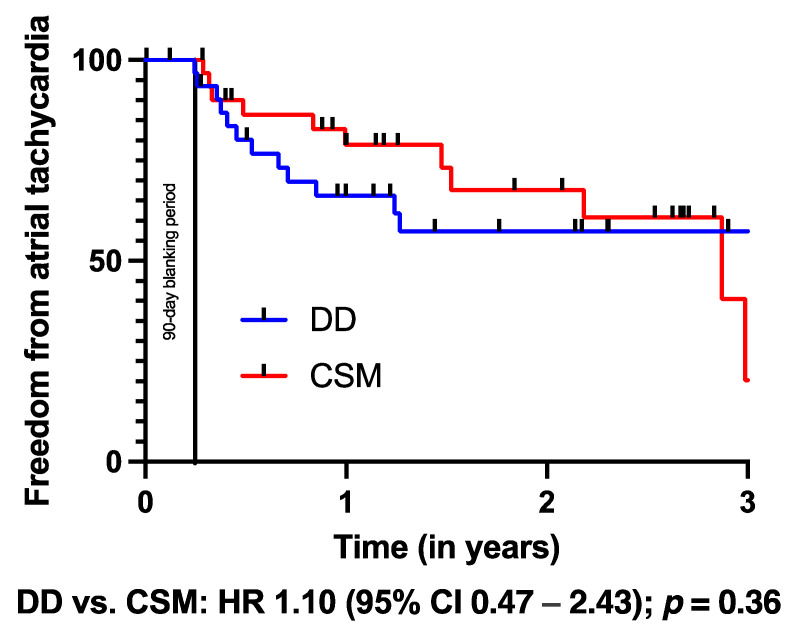
Kaplan–Meier analysis of freedom from atrial tachycardia on or off AADs. A 90-day blanking period was observed.

The Kaplan–Meier analysis (Table 5) revealed a significantly better outcome regarding freedom from any AA after conventional substrate modification (HR: 1.47, *p* = 0.04, Figure 1). Of note, freedom from AF (HR: 0.99, *p* = 0.47, Figure 2) and AT (HR: 1.10, *p* = 0.36, Figure 3) did not differ between both groups.

Within the DD group, a subanalysis demonstrated comparable freedom from atrial arrhythmias during long-term follow-up between patients whose AF terminated into SR through CA (2 of 12 patients (17%)) and those who were cardioverted into SR (3 of 17 patients (18%), HR: 0.88, *p* = 0.86).

Similarly, a further subgroup analysis of the DD group indicated that there were no differences regarding arrhythmia-free survival during long-term follow-up between patients presenting with spontaneous AF (2 of 19 patients (11%)) and those with induced AF (3 of 10 patients (30%), HR: 1.01, *p* = 0.96).

During the long-term follow-up, significantly more redo ablations were performed in the DD group (DD: 16 patients (50%), CSM: 7 patients (22%), *p* = 0.04) during follow-up. Redo CA modalities did not differ between both groups and are listed in Table 6.

## 4. Discussion

The optimal ablation strategy in patients with recurrence of persAF after initially successful PVI is still the subject of ongoing discussions. Our retrospective study provides the first long-term outcome data comparing a DD-guided ablation approach to conventional RF ablation for repeat CA in persAF and reveals no benefit in overall arrhythmia-free survival for DD-guided CA. Furthermore, more repeat procedures were performed in the observation period after DD-guided ablations mostly due to consecutive AT.

Considering the lower success rates for PVI only in the setting of persistent AF, it is presumed that arrhythmogenic substrate outside the pulmonary veins triggers and perpetuates persAF [22]. A DD-guided CA approach aims for a different concept of detectable myocardial cell discharge [20] and, therefore, potentially displays highly resolved AF wavefronts in persAF with fewer farfield annotations as compared to conventional mapping [15]. The first data for DD-guided ablation in patients suffering from persAF and undergoing their first AF ablation were reported in the non-randomized UNCOVER AF study, showing an arrhythmia-free survival rate of 69% after 12 months [13]. Furthermore, DD-guided ablation for the recurrence of persAF was evaluated in the non-randomized RECOVER AF study, reporting an arrhythmia-free survival rate of 54% after 12 months for the first redo AF ablation [17]. Of note, a randomized study by Shi and colleagues compared DD-guided CA to PVI plus PWI in patients undergoing their first AF ablation and reported a potential benefit of DD-guided CA regarding arrhythmia-free survival [14]. However, randomized data comparing DD-guided ablation to conventional substrate modification for repeat persAF ablation are sparse.

In contrast to the UNCOVER AF and RECOVER AF trials [10,14], our single-center study revealed a less favorable outcome after DD-guided CA. Of note, UNCOVER AF and RECOVER AF were both multicenter trials enrolling more than 100 patients, respectively [13,17].

Our data suggest that conventional substrate modification leads to higher arrhythmia-free survival rates and fewer repeat ablations as compared to DD-guided CA in the setting of recurrence of persAF. Although DD-guided ablation assumingly enables a more tailored approach, the endpoint at pivotal ablation sites remains uncertain, and repeat re-mapping with the potential abolishment of AF drivers is the only acute validation option. The obligatory use of a non-contact force catheter (at this time) in DD-guided CA might have contributed to inhomogeneous lesions, therefore leading to an ineffective abolishment of AF drivers and assumingly early, at least partial recovery of ablation lesions, which might be the reason for the trend to more AT ablations after DD-guided CA. 

Of note, in the CSM group, a higher number of PV re-isolations were performed, and significantly more patients underwent PWI. Isolation of the posterior wall is discussed controversially in the literature [23,24]. Incomplete isolation of the latter might contribute to the initiation and perpetuation of AF and/or AT. Recently published data from a meta-analysis supports the hypothesis that additional PWI in persAF might lead to improved arrhythmia-free survival [25]; however, no clear superiority has been demonstrated so far for this approach.

In previous studies, several underlying mechanisms for AF recurrence, such as rotors, focal, and other AF wavefronts, were explored. However, overall outcome data was heterogeneous, and none of the mapping systems was able to show superiority regarding arrhythmia-free survival in these patients [12,26,27]. Recently published data of the RedoFIRM trial comparing FIRM-guided ablation to conventional substrate modification for repeat CA in persAF reported a 12-month arrhythmia-free survival of 54% in the FIRM group and an overall comparable outcome between both groups [28].

In our study, DD-guided CA was associated with longer procedures and fluoroscopy times, indicating a higher level of complexity and radiation exposure for staff and patients. Considering the fact that all procedures were performed under deep analgosedation, the risk of adverse events is enhanced [29]. The necessity of repetitive maps combined with the associated interpretation could be one driver for longer procedure times in the DD group. However, a comparable overall low complication rate reassures that DD-guided CA seems to be equally safe.

Although recently published data demonstrated that an additional tailored low-voltage guided substrate modification in the LA beyond PVI reduces the burden of AA in persAF significantly compared to PVI alone [30] in patients receiving their first AF ablation, there is still a lack of data proving an ablation strategy to be superior for repeat CA in persAF. 

In general, overall success rates, irrespective of the ablation strategy used, are modest, indicating a further need for research to find the optimal ablation strategy in repeat CA for persAF. However, new ablation technologies, such as pulsed-field ablation, might also facilitate durable treatment for persAF.

This study has several limitations. Of note, this trial was a retrospective single-center observation. In contrast to the CSM group, all DD-guided procedures were performed without a contact force-enabled catheter. By now, the system enables contact force catheters, and the overall visualization has improved. Similar to numerous other studies, the evaluation of arrhythmia recurrence primarily relied on 24 h Holter ECG monitoring for most patients. Nonetheless, employing continuous rhythm monitoring methods, such as implantable loop recorders, would have been preferable for a more comprehensive assessment.

## 5. Conclusions

The first data comparing DD-guided CA to conventional substrate modification for repeat CA in persAF revealed significantly longer procedure and fluoroscopy times in the DD group. Long-term outcomes regarding freedom from atrial arrhythmias were better in patients undergoing conventional substrate modification in this single-center observation. Although DD-guided ablation might facilitate a tailored ablation approach for the recurrence of persAF, intraprocedural ablation endpoints at targeted sites remain unclear.

## Figures and Tables

**Table 1:** Baseline characteristics. Values are presented as mean ± SD. Categorical data are given as *n* (%). EHRA: European Heart Rhythm Association. AF: atrial fibrillation. BMI: body mass index. LA: left atrium. LVEF: left ventricular ejection fraction. CAD: coronary artery disease. GFR: glomerular filtration rate. AAD: antiarrhythmic drug. CIED: cardiac implantable electronic device. ICD: implantable cardioverter-defibrillator. CRT: cardiac resynchronization therapy. ILR: implantable loop recorder.

	DD (*n* = 32)	CSM (*n* = 32)	*p* Value
Age, y	65 ± 11	65 ± 9	0.90
Women	8 (25)	8 (25)	1.0
EHRA score	3.5 ± 0.7	3.4 ± 0.7	0.46
Cardioversions	5 ± 4	4 ± 3	0.98
Previous AF ablations	1.8 ± 1.0	1.6 ± 0.8	0.07
BMI, kg/m^2^	28.7 ± 4.6	28.1 ± 3.8	0.29
LA diameter, mm	44.4 ± 6.8	43.7 ± 6.8	0.33
CHA_2_DS_2_-VASc score	2.6 ± 1.3	2.5 ± 1.2	0.64
LVEF, %	53.7 ± 12.3	46.3 ± 13.8	0.38
CAD	6 (19)	7 (22)	1.0
Hypertension	27 (84)	21 (66)	0.15
Diabetes mellitus	2 (6)	4 (13)	0.67
Hyperlipidemia	10 (31)	10 (31)	1.00
GFR, mL/min	71 ± 24	70 ± 21	0.68
AADs	20 (63)	17 (53)	0.62
Amiodarone	13 (41)	11 (34)	0.80
Flecainide	5 (16)	4 (13)	1.0
Dronedarone	2 (6)	0	0.49
Sotalol	0	1 (3)	1.0
CIED	6 (19)	3 (9)	0.47
Pacemaker	2 (6)	0	0.49
ICD	1 (3)	1 (3)	1.0
CRT	1 (3)	1 (3)	1.0
ILR	2 (6)	1 (3)	1.0

**Table 2:** Ablation strategies. Categorical data are given as *n* (%). PV: pulmonary vein. CTI: cavotricuspid isthmus. PWI: posterior wall isolation.

	DD (*n* = 32)	CSM (*n* = 32)	*p* Value
PV re-isolation	16 (50)	21 (66)	0.31
CTI ablation	6 (29)	5 (16)	1.0
Mitral isthmus line	9 (28)	4 (13)	0.21
Roof line	8 (25)	9 (28)	1.0
Anterior line	5 (16)	3 (9)	0.71
Inferior line	2 (6)	0	0.49
PWI	2 (6)	10 (31)	0.02
Defragmentation	32 (100)	23 (72)	0.002

**Table 3:** Rhythm at the start of the procedure. Categorical data are given as *n* (%). SR: sinus rhythm. AF: atrial fibrillation. AT: atrial tachycardia.

		DD (*n* = 32)	CSM (*n* = 32)	*p* Value
Start rhythm	SR	20 (63)	17 (53)	0.61
	AF	10 (31)	15 (47)	0.31
	AT	2 (6)	0	0.49

**Table 4:** Procedural data. Data are presented as mean ± SD. RF: radiofrequency.

	DD (*n* = 32)	CSM (*n* = 32)	*p* Value
Procedure time, min	236 ± 61	198 ± 59	0.004
Fluoroscopy time, min	36 ± 15	20 ± 11	0.0001
Energy, J	123,553 ± 50,664	121,138 ± 49,343	0.98
RF duration, s	4229 ± 1677	3585 ± 1485	0.16
High-frequency impulses	63 ± 28	111 ± 77	0.0004

**Table 5:** Outcome data after CA. Categorical data are given as *n* (%). AA: atrial arrhythmia. AF: atrial fibrillation. AT: atrial tachycardia.

	DD (*n* = 32)	CSM *(n* = 32)	HR	*p* Value
Freedom from AA	6 (19)	11 (34)	1.47	0.04
Freedom from AF	16 (50)	18 (56)	0.99	0.47
Freedom from AT	20 (62)	21 (66)	1.10	0.36

**Table 6:** Modalities of redo CA after initial CA. Categorical data are given as *n* (%). AF: atrial fibrillation. AT: atrial tachycardia.

	DD (*n* = 32)	CSM (*n* = 32)	*p* Value
Total	16 (50)	7 (22)	0.04
Repeat AF ablation	7 (22)	3 (9)	0.30
AT ablation	9 (28)	4 (13)	0.21

## Data Availability

The data presented in this study are available on reasonable request from the corresponding author.
